# Ethnic Differences in Returning Home: Explanations From a Life Course Perspective

**DOI:** 10.1111/jomf.12399

**Published:** 2017-04-18

**Authors:** Tom Kleinepier, Ann Berrington, Lenny Stoeldraijer

**Affiliations:** ^1^ Delft University of Technology and Netherlands Interdisciplinary Demographic Institute/Koninklijke Nederlandse Akademie van Wetenschappen/University of Groningen; ^2^ University of Southampton; ^3^ Statistics Netherlands

**Keywords:** ethnicity, family life, intergenerational relationships, transitions, young adults

## Abstract

Ethnic differences in leaving and returning home may reflect varying cultural norms regarding intergenerational coresidence, but also differences in transitions in linked domains, for example, employment and partnership transitions. This study uses Dutch population register data to compare returning home among second‐generation Turks, Moroccans, Surinamese, and Antilleans with native Dutch who had left the parental home between age 16 and 28 in the period 1999 to 2011 (N = 194,020). All second‐generation groups were found to be more likely to return home than native Dutch. A large part of these differences was related to the timing and occurrence of other key events in the life course, such as age at leaving home and partnership dissolution. Although the impact of partnership dissolution on returning home was found to be strong among all origin groups, it was less pronounced among second‐generation youth, particularly Turks and Moroccans, than native Dutch youth. Possible explanations and implications are discussed.

The transition to residential independence continues to be an important marker of the transition to adulthood (Corijn & Klijzing, 2001). However, this transition has become more protracted and nonlinear in the United States and Europe, with increasing proportions of young adults boomeranging back to the parental home (South & Lei, 2015; Wobma & de Graaf, 2010). Recent studies have sought to explain this trend in terms of broader changes in the life course experiences of young adults, including economic uncertainty arising from precarity in the youth labor market, lack of affordability in the housing market, and the instability of partnerships (Copp, Giordano, Longmore, & Manning, 2015; Sandberg‐Thoma, Snyder, & Jang, 2015; Stone, Berrington, & Falkingham, 2014). Returning home can have negative implications, impacting relationships with parents, peers, and intimate partners (Lewis, West, Roberts, & Noden, 2015; Sassler, Ciambrone, & Benway, 2008). At the same time, moving back to the parental household can also provide financial relief and emotional support to those who have been affected by job loss, housing insecurity, or partnership breakdown (Kaplan, 2012; Lewis et al., 2015). The implications of returning home for young adults and their parents depend on the reason for returning. Extended coresidence has been found to be associated with declines in parent and child well‐being in situations where returning home coincides with negative events such as job loss (Copp et al., 2015; Davis, Kim, & Fingerman, 2016). It is thus important to gain a deeper understanding of the life course events that trigger returns home and how they differ according to individual and parental characteristics.

The implications of returning home also depend on the extent to which extended coresidence is viewed as nonnormative (Davis et al., 2016), which may differ across cultural groups. In many Western European countries, early residential independence and autonomy from parents are highly valued and are well supported by advanced welfare states (Aassve, Arpino, & Billari, 2013). Returning home is generally portrayed negatively in public discourse (Kins & Beyers, 2010). Many non‐Western societies, in contrast, are more collectivistically oriented, which is reflected in strong family ties and intergenerational support obligations (Kagitçibasi, 1996; Nauck, 2002). Although leaving home is not necessarily associated with a break in collectivistic family ties because parents and children can maintain close family links and exchanges while living in separate households, returning is more strongly in contrast with values of individualism and residential independence. Cultural norms may thus particularly affect the frequency and meaning of returning home. International migrants and their offspring form an increasing proportion of the population in many European countries (Eurostat, 2011). It is crucial, therefore, to gain more insight into the patterns of returning home among young people from migrant families who are influenced by the cultural norms held by their parents and the more individualistic society in which they grow up.

Existing studies including racial–ethnic differences in returning home are confined to North America (Britton, 2013; Lei & South, 2016; Mitchell, Wister, & Gee, 2004). This article provides first insights for Europe, examining ethnic variation in returning home among young adults living in the Netherlands. We focus on the second generation of the four largest non‐Western immigrant groups in the country (Turks, Moroccans, Surinamese, Antilleans) and native Dutch. The Netherlands is a valuable case study given its ethnically diverse population composition with differing norms and values regarding intergenerational coresidence (de Valk & Liefbroer, 2007b). Contrary to the generally late age at leaving home in the origin countries, migrant youth in the Netherlands leave the parental home at younger ages than native Dutch youth, which has been suggested to relate to higher levels of conflict in migrant families (Zorlu & Mulder, 2011). However, leaving home earlier does not necessarily mean that family bonds are neglected: Collectivistic family ties may be more important for returns to the parental home.

To study the mechanisms underlying ethnic differences in returning home, a life course framework is needed that emphasizes how earlier life events impact those that occur in later life (Giele & Elder, 1998). Ethnic differences in the timing of leaving home may strongly affect subsequent home‐returning behavior. Furthermore, previous research has highlighted the importance of turning point events in the life course (e.g., losing a job, union dissolution) as predictors of home returning (Stone et al., 2014). The timing and frequency of these turning points also differ between ethnic groups. For instance, second‐generation migrants in the Netherlands are generally less successful than natives in the labor market (van der Vliet, Ooijevaar, & Wobma, 2014). Life course transitions in other domains may thus affect the association between ethnicity and home returning. In addition, the impact of partnership transitions on returning home might differ by ethnic group: Qualitative research suggests that Turkish and Moroccan youth may be less likely to rejoin the parental home after divorce because their parents deem divorce as socially unacceptable (Sterckx & Bouw, 2005). Ethnicity may thus moderate the impact of partnership dynamics on returning home. This study seeks answers to the following three research questions: To what extent are there differences in the likelihood of returning to the parental home between Turkish, Moroccan, Surinamese, and Antillean second generation and native Dutch young adults? To what extent are differences in the timing and occurrence of key life events related to the relationship between ethnicity and returning home? To what extent does ethnicity moderate the relationship between partnership dynamics and returning home?

To address our research questions, we use administrative micro data that cover the entire population of the Netherlands (Bakker, van Rooijen, & van Toor, 2014). The data contain detailed information on the occurrence and timing of leaving and returning to the parental home as well as partnership, education, and work histories. Our analysis includes every registered person of the Turkish, Moroccan, Surinamese, and Antillean second generation and a 5% random sample of native Dutch youth who had left the parental home between the ages of 16 and 28 in the 1999–2011 period (*N* = 194,020). Discrete‐time hazard models are employed to test a series of hypotheses relating to ethnic differences in returning home.

## Background

### *Migrants in the Netherlands*

About one fifth of the 17 million inhabitants of the Netherlands has a foreign background—that is to say, has at least one parent born abroad, including those born abroad themselves (first generation) and those born in the Netherlands (second generation). The four largest non‐Western origin groups (Turks, Moroccans, Surinamese, Antilleans) comprise, respectively, 2.3%, 2.3%, 2.1%, and 0.9% of the current total Dutch population (Statistics Netherlands, 2016). Many Turkish and Moroccan immigrants, initially recruited as temporary low‐skilled laborers during the 1960s, settled permanently in the Netherlands and were joined by their families in the 1970s and 1980s (Vermeulen & Penninx, 2000). Subsequently, many had children born in the Netherlands, and slightly more than half of the Turkish and Moroccan population in the Netherlands is now of the second generation. The vast majority of the Turkish (78%) and Moroccan (82%) second generation has two parents born abroad (Statistics Netherlands, 2016).

Immigration flows from Surinam and the (former) Netherlands Antilles relate to Dutch colonial history, and most of these immigrants were therefore familiar with the Dutch language and culture upon arrival (Oostindie, 2011). Shortly before Surinam gained its independence in 1975, many Surinamese migrated to the Netherlands to retain Dutch citizenship. Immigration from the Antilles peaked in the 1980s when economic conditions on the islands worsened. In contrast to the predominantly Muslim Turkish and Moroccan migrants, Surinamese and particularly Antillean migrants are primarily Christian (van Tubergen, 2003). Slightly less than one half of the Surinamese and Antillean population in the Netherlands are classified as second generation (Statistics Netherlands, 2016). About 38% of the Surinamese and 56% of the Antillean second generation has one parent born in the Netherlands, reflecting the relatively high out‐partnering rate among these origin groups (Kalmijn & van Tubergen, 2006; Statistics Netherlands, 2016).

All four groups have a disadvantaged socioeconomic position, but Turks and Moroccans experience a larger gap in educational attainment and labor market outcomes with respect to the native Dutch than do Surinamese and Antilleans (van der Vliet et al., 2014). Furthermore, the four origin groups are concentrated in urbanized areas, with around 56% living in the four largest cities (Amsterdam, Rotterdam, The Hague, and Utrecht), compared to 15% of native Dutch (Statistics Netherlands, 2016).

### *Housing and Welfare Benefits*

It has been argued that the availability of affordable housing and welfare entitlements go some way to explain the relatively early home leaving of ethnic minority youth in the Netherlands (Zorlu & Mulder, 2011). In comparison with other European countries, social housing in the Netherlands is generally available (making up one third of the total housing stock; Statistics Netherlands, 2016) and affordable (being subsidized by the state and subject to rent control). For example, tenants living alone with a gross annual income below €22,100 and tenants living with a partner or housemate with a combined gross annual income below €30,000 can receive housing allowance as long as they or their partner do not have savings of more than €24,437 (http://www.belastingdienst.nl/english). Unemployment benefits, student loans, and healthcare allowances further allow young adults to live independently from parents, regardless of income or ethnic background (Kleinepier & de Valk, 2016; Zorlu & Mulder, 2011). The fact that native Dutch young people choose to leave home somewhat later than second‐generation youth has recently been explained by the fact that the parents of the native Dutch youth will on average be in a more comfortable housing situation than the young person would be able to achieve if he or she left home. In contrast, there is more of an incentive for the second generation to leave home because they will on average experience an upward move in housing quality and privacy if they leave (Zorlu & van Gaalen, 2016).

## Theory and Hypotheses

### *Individualism–Collectivism and Conflict*

Young adults' residential careers will be affected by culturally specific values on the role assigned to the individual versus the role of wider kin and social groups, which have been found to differ by ethnicity (Kagitçibasi, 1996; Phalet & Schönpflug, 2001). The following two broad contrasting cultural orientations can be found: individualistic, with a strong emphasis on independence, autonomy, and self‐development, and collectivistic, where interdependence, loyalty, and the needs of the in‐group are pivotal (Hofstede, 1980; Kagitçibasi, 1996). The Netherlands is a society in which a more individualistic orientation prevails (Oppenheimer, 2004). Parents spend a great deal of time teaching and encouraging their children to become and remain independent and self‐sufficient adults (Keller, Borke, Yovsi, Lohaus, & Jensen, 2005). The proclivity for a return to the parental household is expected to be rather low in a social context where (residential) independence is valued because returning home may be perceived as the inability to function independently (South & Lei, 2015).

Conversely, Turkey, Morocco, Surinam, and the (former) Netherlands Antilles are more collectivistically oriented, with greater importance placed on kinship ties and family obligation (Kagitçibasi, 1996; Phalet & Schönpflug, 2001). In these societies, multigenerational households of adult children living with their parents are common, even when the children have a coresidential partner or children of their own (Nauck, 2002). Because the parents of the second generation grew up and were socialized primarily in the countries of origin, we assume that they were more family oriented prior to immigration to the Netherlands. In turn, parents exert a strong normative influence on their children (de Valk & Liefbroer, 2007b), and we expect that the second generation's attitudes are therefore marked by a pronounced inclination toward collectivism over individualism as well. Moreover, the migration process itself may have strengthened family ties because families provide an important source of orientation and support after moving to a new society (Pyke, 2003). Leaving home does not necessarily mean that family ties are neglected, because children and their parents can still support each other when living in separate places. Returning to the parental home may, however, jeopardize the ideal of being independent and autonomous. We therefore expect that the individualism–collectivism cleavage is particularly relevant for home‐returning behavior and hypothesize the following: Young adults of the Turkish, Moroccan, Surinamese, and Antillean second generation are more likely to return home than those of native Dutch origin (Hypothesis 1a).

Rather than strengthening family ties, international migration has also been shown to threaten the harmony and stability of family relations (Phalet & Schönpflug, 2001), particularly for the second generation, who are exposed to alternative cultural values and contrasting ways of thinking through contact with peers, the media, and school (Huschek, Liefbroer, & de Valk, 2010). Previous research suggests that cultural distance between the culture of origin of the parents and that of the society in which the children grow up may result in intergenerational tensions and can create strains in migrant families (Giguère, Lalonde, & Lou, 2010; Lou, Lalonde, & Giguère, 2012). Indeed, Zorlu and Mulder (2011) argue that intergenerational tensions resulting from culture clash might be an important factor in encouraging early home leaving among Turkish and Moroccan youth in the Netherlands. Previous research indicates that family conflict or low intergenerational closeness also discourages returning to the parental home (Goldscheider & Goldscheider, 1998). In view of these theoretical propositions and empirical findings, we may thus expect that the children of immigrants are less likely to return to live with their parents. Accordingly, we derive the following hypothesis: Young adults of the Turkish, Moroccan, Surinamese, and Antillean second generation are less likely to return home than those of native Dutch origin (Hypothesis 1b).

### *The Role of Life Course Transitions*

Ethnic differences in returning home may further be related to differences in the timing and occurrence of other key events in the life course. Individuals who leave home at younger ages are much more likely to return (Goldscheider & Goldscheider, 1998; Stone et al., 2014). There are a number of reasons for this. First, young home‐leavers may depart with little or no means of support and may not be ready to live independently (Mitchell et al., 2004). Second, returning home may be more socially acceptable at younger ages because of (implicit) age norms on parent–child coresidence (Aassve et al., 2013; Stone et al., 2014). Third, reasons for leaving home vary strongly with age at departure; those who leave home at older ages more frequently start living with a partner (Jones, 1995). Recent findings suggest that migrant youth, particularly Turks and Moroccans, leave home at significantly younger ages and more often to live alone independently than the native Dutch (Kleinepier & de Valk, 2016; Zorlu & Mulder, 2011). We therefore expect the following: Young adults who have left the parental home at young ages are more likely to return to the parental home (Hypothesis 2a). Differences in returning home between the Turkish, Moroccan, Surinamese, and Antillean second generation and those of native Dutch origin become attenuated after accounting for the timing of leaving the parental home (Hypothesis 2b).

Path interdependency within the life course emphasizes the importance of other, parallel careers in conditioning and triggering the choice to return to the parental home (Giele & Elder, 1998; Stone et al., 2014). The residential career is particularly intertwined with the labor market and family trajectory (Corijn & Klijzing, 2001). Being or becoming unemployed and failing to find a job after college graduation increase the need for parental support and, hence, are important drivers of moving back to the parental home (Davanzo & Goldscheider, 1990; Kaplan, 2012; South & Lei, 2015; Stone et al., 2014). We argue that differences in economic activity will relate to ethnic differences in returning home. When compared with the native Dutch population, the four second‐generation groups under study are more likely to experience unemployment and to make an unsuccessful transition from school to work (van der Vliet et al., 2014). Unemployment rates among recent graduates from lower tertiary education are much higher among the Turkish (20%), Moroccan (26%), Surinamese (17%), and Antillean (28%) second generations than among the native Dutch population (5%; Meng, Verhagen, & Huijgen, 2013). Therefore, we come to the following two hypotheses: Being or becoming unemployed increases the likelihood of returning to the parental home (Hypothesis 3a). Differences in returning home between the Turkish, Moroccan, Surinamese, and Antillean second generation and those of native Dutch origin become attenuated after accounting for economic activity (Hypothesis 3b).

Entry into a partnership increases young adults' preferences for privacy and residential independence (Smits, van Gaalen, & Mulder, 2010), reducing the tendency to return home (South & Lei, 2015; Stone et al., 2014). Partnership dissolution often leads to a decrease in financial resources, for instance, through the division of joint ownership of spouses or alimony payments, and increases the propensity of returning home (South & Lei, 2015; Stone et al., 2014). Recent empirical research indicates that the second‐generation groups are more likely to live independently without a partner throughout young adulthood than are the native Dutch (Kleinepier & de Valk, 2016; Zorlu & van Gaalen, 2016). With regard to partnership dissolution, the percentage of married couples who divorce within 10 years of marriage is higher among the Turkish (21%), Moroccan (16%), Surinamese (27%), and Antillean (16%) second generation than among the native Dutch (12%; Smith, Maas, & van Tubergen, 2012). Part of the explanation for this difference is the higher level of cohabitation among native Dutch young adults, which often acts as a testing phase before marriage (Hiekel & Keizer, 2015). Rooyackers, Das, and de Valk (2015) show that overall levels of union dissolution (including both cohabitation and marital dissolution) are similar for the Turkish and Moroccan second generation and the native Dutch, but are higher for the Surinamese and Antillean second generation. Given these patterns we expect the following: Young adults living unpartnered and those who experience partnership dissolution are more likely to return to the parental home (Hypothesis 4a). Differences in returning home between the Turkish, Moroccan, Surinamese, and Antillean second generation and those of native Dutch origin become attenuated after accounting for partnership dynamics (Hypothesis 4b).

### *Partnership Dynamics by Ethnic Origin*

In Turkish and Moroccan societies, high importance is placed on marriage, and divorce is traditionally considered to be socially unacceptable (Nauck, 2002). Although tolerance toward divorce has increased in Turkey, divorcees still feel that they are blamed (Kavas & Gündüz‐Hoşgör, 2010). Despite observed high divorce rates for the Turkish and Moroccan second generation in the Netherlands, qualitative research suggests that they, too, perceive considerable disapproval of divorce from their parents and families (Hooghiemstra, 2003; Sterckx & Bouw, 2005). The strong preference for marriage among Turkish and Moroccan parents is accompanied by an opposition to unmarried cohabitation (de Valk & Liefbroer, 2007a). Furthermore, the family life behavior of daughters is more strongly supervised than that of sons because women in particular may put a family's reputation and honor at risk through disapproved actions (Nauck, 2002; Phalet & Schönpflug, 2001).

Family life in the Caribbean area is characterized by unmarried cohabitation with high union instability, single‐mother households, and multipartner fertility (Shaw, 2003). Surinamese and Antillean migrants in the Netherlands are much more likely to accept unmarried cohabitation than Turks and Moroccans (de Valk & Liefbroer, 2007b). Among native Dutch, the institution of marriage has weakened during the past decades, whereas the moral acceptance of cohabitation and divorce has increased substantially (Corijn & Klijzing, 2001; Lesthaeghe, 2010). We expect that the greater disapproval of cohabitation and divorce among the parents of the Turkish and Moroccan second generation will make them reluctant to welcome their children back home after partnership dissolution, particularly with regard to daughters. Therefore, we formulate the following two hypotheses: The impact of partnership dissolution on returning home is weaker for the Turkish and Moroccan second generation than for the native Dutch (Hypothesis 5a). This interaction effect is particularly evident among women (Hypothesis 5b).

### *Additional Factors Influencing Returning Home*

There are several other determinants that may account for ethnic differences in returning home. We briefly describe these factors, which mainly serve as control variables. Becoming a parent strongly increases the likelihood of leaving home (South & Lei, 2015), but its relationship with returning home has been found, at least in the U.K. context, to be moderated by gender and partnership status: Fathers are more likely to return home than mothers following partnership dissolution (Stone et al., 2014). Hence parenthood is included as a control and interacted with gender. We also control for the young adult's educational attainment, because those with a higher education have more resources to establish and maintain residential independence (Blaauboer & Mulder, 2010; Sandberg‐Thoma et al., 2015).

Parents with higher incomes can afford to assist their children with the costs of independent housing, for example, in the form of a rental or mortgage deposit (Stone et al., 2014). Yet parental resources may also serve as a proxy for the quality and attractiveness of the parental home environment that may affect the likelihood of returning home (Ermisch, 1999). This “feathered‐nest” hypothesis has received inconsistent empirical support (Mulder & Clark, 2002, p. 984). We include the occupational status of the father and mother as a proxy for parental resources. Children whose biological parents do not live together are found to leave home earlier and to be less likely to return home, possibly because of problematic parent–child relationships resulting from changes in the family structure (Goldscheider & Goldscheider, 1998). Moreover, the union status of parents dictates parental socioeconomic resources available to children (Aquilino, 1991). We therefore control for parental union status.

Young adults' incentives to return home are greater when the parents live in an urban area because of more educational and job opportunities near the parental home (Mulder & Clark, 2002; South & Lei, 2015). Similarly, young adults may be less willing to return home if their own independent residence is located in an urban area. Therefore, we account for the urbanicity of the area where the parental home and the young adult's place of residence are located. Finally, the number of young adults returning home in the Netherlands has increased substantially during the past decade (Wobma & de Graaf, 2010). Because this may relate to increased economic uncertainty, we distinguish between four periods in terms of distinct breaks in the youth unemployment rate in the Netherlands, including the 2008–2011 recession.

## Method

Our analyses are based on the System of Social Statistical Datasets (Bakker et al., 2014). This dataset was constructed by Statistics Netherlands in the late 1990s by matching multiple administrative registers that cover the entire population of the Netherlands, including the Dutch municipal population register and tax registers. We avoid problems of left censoring by selecting those who first left the parental home (while aged 16–28 years) between January 1999 and November 2011 and thus became at risk of returning home. This age range is chosen because the vast majority (more than 94%) of young adults from all ethnic groups had left home between these ages. We select all persons of the Turkish (*n* = 39,904), Moroccan (*n* = 32,527), Surinamese (*n* = 32,128), and Antillean (*n* = 8,678) second generation, along with a 5% random sample of the native Dutch (*n* = 80,783) corresponding to the aforementioned criteria.

### Measures

#### *Returning home*

Individuals are matched to their biological or adoptive mother and father using unique individual registration numbers. People are classified as living in the parental home if they are registered at the same address as at least one biological or adoptive parent. Address information was available on a monthly basis. The dependent variable is a binary indicator of whether a person moved into the address of at least one of his or her parents at each month of observation (0 = “no,” 1 = “yes”). Cases where the parent(s) had moved in with the young adult and shared moves of both generations are not considered returns to the parental home.

#### *Origin group*

A person is classified as second‐generation Turkish, Moroccan, Surinamese, or Antillean if he or she was born in the Netherlands and has at least one parent who was born abroad. If both parents were born abroad, but in different countries, the country of birth of the mother is dominant. Dutch population registers include information on parents' country of birth also when they are living abroad. Those with two native‐born parents are classified as native Dutch, irrespective of their own birth country. In cases where the country of birth of the father is unknown, it is assumed to be the same as the country of birth of the mother (and vice versa). Mixed parentage is a dummy variable that denotes whether the young adult has one foreign‐born parent and one native‐born parent (0 = “no,” 1 = “yes”).

*Age at first leaving the parental home* is time‐constant and grouped: 16–18 (reference), 19–21, 22–24, and 25–28. Similar to previous studies (e.g., Davanzo & Goldscheider, 1990; South & Lei, 2015; Stone et al., 2014), we capture life course stages and transitions with dummy variables indicating a change or nonchange in circumstances at each month when compared with the previous month. For example, being employed at *t* and unemployed at *t*−1 is classified as from unemployed to employed at time *t*. This implies that we estimate only the instantaneous effect of the turning points on returning home. Because some events can also have a delayed or lagged effect on returning home, we carried out sensitivity analysis using 3‐month, 6‐month, and yearly intervals, all of which yielded substantially similar results (available on request). *Economic activity* is captured by the following eight dummy variables: stable employed (reference), stable unemployed, stable student, from student to employed, from student to unemployed, new student, from employed to unemployed, and from unemployed to employed. Statistics Netherlands classifies individuals who are both in education and employed as “students” if they earn less than the low‐income threshold and as “employed” otherwise. The low‐income threshold is based on the level of social assistance benefit for a single person in 1979 and is adjusted yearly for inflation. The vast majority of students earn less than the low‐income cut‐off. *Partnership dynamics* are coded into the following six categories: stable unpartnered (reference), stable cohabiting, stable married, new cohabiting, new marriage (including from cohabiting to marriage), and partnership dissolution. The rare transition from marriage to cohabitation (living together after legal divorce) is not coded as a turning point. Furthermore, because of the low frequency of marital divorce, we do not distinguish marital and cohabitation dissolution. Finally, to keep the number of partnership status categories within manageable limits, we do not distinguish between individuals living alone and those sharing accommodation with adult others.

We include several control variables. *Gender* is a dummy variable (female = 0, male = 1). *Parenthood* indicates whether the young adult coresides with at least one child younger than age 18 at each monthly time interval (0 = “no,” 1 = “yes”). *Educational level* is measured with a time‐varying binary variable indicating whether the person has obtained a degree in higher education in the Netherlands (0 = “no,” 1 = “yes”). Dutch higher education comprises university education and higher vocational education. A degree in higher vocational education is equivalent to a bachelor's degree from, for example, a British, American, or Canadian university. We employ this relatively crude distinction between higher and lower education because administrative registers on secondary and lower tertiary education are only available for the more recent birth cohorts. *Living in an urban area* is a time‐varying dummy variable on whether the young adult was living in the highly urbanized “Randstad” region of the Netherlands (0 = “no,” 1 = “yes”). *Parental home in an urban area* is a similarly coded variable indicating if the mother, father, or both parents lived in the Randstad area. *Parental union status* is a dummy variable that indicates on a monthly basis whether the young adult's parents were living together (0 = “no,” 1 = “yes”). *Parental occupational status* is captured with two dummy variables that measure the employment status of the father and mother on a monthly basis (0 = not employed, 1 = employed). The relatively few cases in which the mother (1.7%) or father (4.3%) was not alive or living in the Netherlands were assigned a value of zero because coding them separately did not produce substantially different results. *Calendar period* is measured by a set of time‐varying dummy variables indicating the period in which the observation occurred: 1999–2001 (reference), 2002–2004, 2005–2007, and 2008–2011.

### *Analytic Strategy*

Discrete‐time logistic event history models are used to estimate the odds that an individual returns home at time *t*, provided he or she did not return home at *t*−1 (Allison, 1984). We construct person‐month files with separate records for each month that an individual was at risk of returning home, starting when the young adult first moves out of the parental home. Repeated spells are not considered: Once having moved back, the individual is no longer observed. The observation is censored after whichever of the following occurs first: (a) the young adult died or emigrated, (b) the young adult's father and mother were both not alive or registered in the Netherlands, (c) at least one parent had moved in with the young adult, (d) both the young adult and parents moved toward a shared address, or (e) the young adult was still living outside of the parental home in the last month of observation, December 2011. Because the last potential month of observation is the same for each individual regardless of when they left home, individuals who left home in earlier calendar periods can be exposed to the risk of returning at longer durations. We therefore undertook sensitivity analyses censoring all observations after 5 years. The results were unchanged, reflecting the fact that most returning home happens soon after initial departure. For instance, about 25% of the total sample had returned home within 5 years when compared with about 29% within 10 years of observation. The baseline hazard is specified as a piecewise constant with six intervals of duration since leaving the parental home: up to 12 months, 13–24 months, 25–36 months, 37–60 months, 61–84 months, and more than 84 months.

The results testing Hypotheses 1 through 4 are presented in six models. Model 1 includes only the dummy variables for the different origin groups and the baseline hazard to show the overall (unadjusted) patterns. In Model 2, the control variables are included. Models 3 to 6 add the life course transition variables to assess the extent to which they attenuate associations between origin group and returning home. Each variable is first entered one by one in Models 3 to 5 to isolate possible indirect effects, then altogether in Model 6 to account for associations among these variables. In following this approach, it is important to recognize that log‐odds ratios and odds ratios depend both on effect sizes and the magnitude of unobserved heterogeneity (Mood, 2010). Consequently, logit coefficients of the same variable in nested models with different covariates are not directly comparable. Karlson, Holm, and Breen (2012) have proposed a method to overcome this problem by rescaling the coefficients. We apply this method using the user‐generated Stata command *khb* (Kohler & Karlson, 2011). For testing Hypothesis 5a, we run the full model for men and women separately and include interaction terms of partnership status with origin group. Finally, we test Hypothesis 5b with a pooled model using a three‐way interaction between partnership status, ethnic origin group, and gender.

### *Sample Characteristics*

Descriptive statistics for all variables used in the analysis by origin group are provided for the first month of living independently (*t*1) and thus tell us about the characteristics of young adults for the month when they first left home (Table 1). The second generation, especially Turks and Moroccans, were much younger than the native Dutch when they left the parental home. The majority of second‐generation youth were still in education when they left (stable student), whereas the native Dutch were mostly enrolled in the labor market (stable employed). This was associated with the different ages at departure, but also because of the longer time on average spent in education of the second‐generation youth when compared with the native Dutch—owing mainly to repeating classes and stacking degrees (Kleinepier & de Valk, 2016; van der Vliet et al., 2014). Separate descriptive analysis by gender (not shown) indicated that women tended to be younger than men when they first left home, apart from the Moroccan origin group, among whom the two genders had a rather similar distribution of age at leaving. Again owing to their younger ages at departure, Turkish and Moroccan second‐generation youth in particular were more frequently living without a partner upon home leaving than the native Dutch. However, Turkish and Moroccan youth were more often married when they left the parental home than the native Dutch. Additional analysis (not shown) indicated that this difference was mainly because of the tendency for Turkish and Moroccan women to be married on leaving home; Moroccan men were even slightly less frequently married at *t*1 than were native Dutch men, a finding that corroborated with Zorlu and Mulder (2011).

**Table 1:** Percentual Distribution of Independent Variables at t1 (Month First Left Parental Home), by Origin Group

	Turkish	Moroccan	Surinamese	Antillean	Dutch
	(*n* = 39,904)	(*n* = 32,527)	(*n* = 32,128)	(*n* = 8,678)	(*n* = 80,783)
Mixed parentage	5.0	6.5	26.5	69.6	N/A
Age at leaving home					
16–18	49.9	43.6	21.6	16.1	9.9
19–21	31.3	37.7	38.1	41.4	32.7
22–24	11.9	12.5	24.3	26.4	32.6
25–28	6.9	6.2	15.9	16.1	24.9
Economic activity					
Stable employed	21.3	18.9	35.6	38.3	57.7
Stable unemployed	5.8	6.5	8.3	6.5	3.7
Stable student	66.5	68.5	48.9	46.0	30.8
Student, employed	1.6	1.8	1.9	2.1	2.3
Student, unemployed	0.9	0.7	1.0	1.2	1.0
New student	2.2	2.2	2.3	4.2	3.2
Employed–unemployed	0.9	0.7	1.2	1.0	0.7
Unemployed–employed	0.8	0.7	0.9	0.7	0.6
Partnership dynamics					
Stable unpartnered	82.4	84.7	69.9	68.1	55.2
Stable cohabiting	0.2	0.0	0.8	0.7	1.0
Stable married	7.4	5.2	2.7	1.0	1.6
New cohabiting	8.7	9.1	25.6	29.6	39.0
New marriage	1.1	0.7	0.7	0.4	3.1
Dissolution	0.3	0.1	0.4	0.2	0.0
Control variables					
Male	48.0	46.2	44.5	45.9	48.4
Child younger than age 18 in household	8.9	5.0	7.4	5.6	2.1
Graduated from higher education	1.7	2.1	5.9	7.8	12.3
Lives in urban area	58.2	70.6	80.7	62.9	40.9
Parental home in urban area	57.0	71.4	81.6	63.3	41.0
Parents live together	76.9	79.7	40.6	52.6	77.7
Father employed	43.5	27.7	52.2	60.4	76.6
Mother employed	24.3	15.3	60.3	60.8	59.8

*Note*. Percentages may not total 100 because of rounding. Percentages for calendar period not presented for reasons of space. N/A, not applicable.

In Table 2, we provide the percentage of young adults who experienced life course stages and transitions by origin group aggregated at the person level. As shown in the table, native Dutch youth returned to the parental home the least frequently of all origin groups. Turkish second‐generation youth were on average the most likely to move back home, followed by the Surinamese, Moroccan, and Antillean second generation. Dutch young adults more often experienced stable employment and were less likely to be in full‐time education or to have had a spell of unemployment. Additional analysis summarizing across all person months (not shown) demonstrated that the duration in these states also differed strongly between ethnic groups. For instance, the percentage of the total observed person‐months spent in unemployment was more than twice as high (at around 12%–14%) for the Turks, Moroccans, and Surinamese when compared with the native Dutch (6%).

**Table 2:** Percentage of Young Adults Who Ever Experienced Life Course Stage or Transition During the Observation Period, by Origin Group

	Turkish	Moroccan	Surinamese	Antillean	Dutch
	(*n* = 39,904)	(*n* = 32,527)	(*n* = 32,128)	(*n* = 8,678)	(*n* = 80,783)
Returning home	38.8	31.2	35.0	25.6	20.4
Economic activity					
Stable employed	61.4	61.5	68.3	68.0	80.3
Stable unemployed	32.7	35.5	33.6	28.0	22.6
Stable student	74.5	76.9	58.8	58.9	39.9
Student, employed	37.8	40.4	30.7	29.3	22.9
Student, unemployed	16.4	17.2	12.1	11.1	6.1
New student	28.5	30.3	24.4	25.6	17.2
Employed–unemployed	26.9	28.0	28.0	23.6	23.1
Unemployed–employed	27.1	27.0	27.8	23.7	22.9
Partnership dynamics					
Stable unpartnered	87.6	90.5	81.2	80.3	65.7
Stable cohabiting	21.2	22.3	48.5	55.8	64.2
Stable married	27.6	24.5	12.0	11.0	24.4
New cohabiting	23.6	25.1	50.7	58.8	65.7
New marriage	20.7	19.9	9.5	10.2	23.1
Dissolution	18.0	21.3	33.2	35.1	29.1

*Note*. Percentages do not total 100 because people may experience multiple states and transitions.

Further corroborating the results in Table 1, we found that the native Dutch experienced living alone independently the least often. The second‐generation groups, particularly Turks and Moroccans, experienced starting or being in a cohabiting relationship much less often than the native Dutch. Whereas Surinamese and Antilleans were also less often in a stable married relationship, the Turkish and Moroccan youth were slightly more often married than the native Dutch. However, the Turkish and Moroccan youth less often experienced the transition to marriage, resulting from the fact that Turkish and Moroccan youth were more often married prior to leaving the parental home (Table 1). Finally, we found that the Surinamese and Antilleans experienced partnership dissolution more often than the native Dutch, whereas the Turks and Moroccans experienced the least often a break‐up at some point during the observation.

## Findings

Table 3 shows the results of the discrete‐time event history models. The coefficients associated with ethnic origin increased with the inclusion of the control variables in Model 2, indicating a suppression effect. Additional analysis (not shown) showed that mainly the occupational status of parents was suppressing the ethnic group differences in Model 1. It is important to note that Models 3 through 6 should be compared with Model 2 because the control variables were added in all of these models. Overall, our findings provide strong support for Hypothesis 1a (and contradict Hypothesis 1b) because all second‐generation groups were significantly more likely to return to the parental home than the native Dutch, even in the model with the most complete set of control and attenuating variables (Model 6).

**Table 3:** Odds Ratios From Discrete‐Time Event History Models of Returning to the Parental Home

	Model 1	Model 2	Model 3	Model 4	Model 5	Model 6
Origin group						
Turkish	2.19***	2.75***	1.93***	2.48***	1.98***	1.72***
Moroccan	1.95***	2.40***	1.66***	2.14***	1.62***	1.37***
Surinamese	2.18***	2.44***	2.04***	2.25***	1.80***	1.69***
Antillean	1.72***	1.92***	1.58***	1.77***	1.37***	1.31***
Dutch (ref.)	1.00	1.00	1.00	1.00	1.00	1.00
Mixed parentage	0.77***	0.68***	0.74***	0.70***	0.74***	0.76***
Age at leaving home						
16–18 (ref.)			1.00			1.00
19–21			0.94***			0.97**
22–24			0.50***			0.63***
25–28			0.26***			0.35***
Economic activity						
Stable employed (ref.)				1.00		1.00
Stable unemployed				1.75***		1.23***
Stable student				1.26***		0.62***
Student, employed				3.33***		1.77***
Student, unemployed				4.29***		2.20***
New student				1.67***		0.84***
Employed–unemployed				2.91***		2.11***
Unemployed–employed				2.75***		2.04***
Partnership dynamics						
Stable unpartnered (ref.)					1.00	1.00
Stable cohabiting					0.03***	0.03***
Stable married					0.08***	0.09***
New cohabiting					0.30***	0.29***
New marriage					0.21***	0.21***
Dissolution					28.43***	27.69***
Control variables						
Male		1.08***	1.20***	1.08***	0.99	1.02*
Child younger than age 18 in household		0.51***	0.58***	0.47***	0.85***	0.78***
Male × Child in household		1.25***	1.38***	1.43***	2.10***	2.31***
Graduated from higher education		0.94***	1.17***	1.00	1.06***	1.16***
Lives in urban area		0.74***	0.73***	0.74***	0.67***	0.67***
Parental home in urban area		1.16***	1.19***	1.16***	1.24***	1.24***
Parents live together		0.87***	0.90***	0.89***	0.96***	1.03**
Father employed		1.32***	1.26***	1.32***	1.34***	1.33***
Mother employed		1.35***	1.31***	1.35***	1.31***	1.32***
Pseudo‐*R* ^2^	0.03	0.04	0.05	0.04	0.16	0.17
Degrees of freedom	10	22	25	29	27	37
No. of observations	*N* person months 11,459,546 | *N* persons 194,020					

*Note*. Controls for baseline hazard function and calendar period are included but not shown for reasons of space. Differences in coefficients across nested models were formally tested using the Stata command *khb* (Karlson et al., 2012). ref. = reference.

* *p* < .05.

** *p* < .01.

*** *p* < .001.

Consistent with Hypothesis 2a, we found a strong negative effect of age at leaving home (especially for leaving at age 22 and older) on the likelihood of returning home. Once age at first leaving home was included in Model 3, the coefficients associated with ethnic origin decreased in magnitude when compared with Model 2 (particularly the estimates for the Turkish and Moroccan second generation), confirming Hypothesis 2b. We also found support for Hypothesis 3a, which predicted that being or becoming unemployed increased the likelihood of returning home: Those who became unemployed either after employment or leaving full‐time education were significantly more likely to return home than those who remained employed at both time points (Model 4). The results further indicated that becoming employed after unemployment or full‐time education also increased the likelihood of returning home. These findings were consistent with previous U.K. research that indicated that successful life course transitions can also precipitate a return to the parental household (Stone et al., 2014). As expected (Hypothesis 3b), ethnic differences in returning home became smaller after accounting for economic activity. Those who experienced partnership dissolution were much more likely to return home than those who remained unpartnered (Model 5), which is in support of Hypothesis 4a. Further in line with this hypothesis, our findings indicated that persons who remained in (or started) a partner relationship were less likely to return home than those who remain unpartnered. Finally, Hypothesis 4b stated that the differences between the Turkish, Moroccan, Surinamese, and Antillean second generation and the native Dutch in returning home are related to different partnership dynamics. The results indicated a decrease in all ethnic group differences in the likelihood of returning home after we controlled for partnership status, thereby lending support to our hypothesis.

We used the Karlson‐Holm‐Breen (KHB) method (Karlson et al., 2012) to estimate the unbiased change in ethnic group differences across Models 2 to 6 in Table 3. The results (available on request) showed that each of the life course variables significantly attenuated the coefficients associated with ethnic origin in Model 2, although the decrease as a result of economic activity was modest. Specifically, including economic activity in Model 4 was found to reduce ethnic group differences by 8% to 12% (depending on ethnic group) as compared to a decrease of 25% to 50% after controlling for age at leaving home in Model 3 and a 54% to 70% decrease after including partnership dynamics in Model 5. Controlling for all life course variables simultaneously (Model 6) provided modest added value over controlling for age at leaving home and partnership status individually, reflecting the strong relationship between the timing of leaving home and partnership behavior: Those who left home at younger ages were less likely to have left home to start living with a partner and vice versa. Moreover, the impact of starting full‐time education (new student) on returning home changed from positive in Model 4 to negative in Model 6. This can be explained by the fact that people who started education were more likely to have left the parental home at younger ages and to be living without a partner. Once these variables were included, the direction of the effect of starting full‐time education reversed, highlighting the interconnectedness of education, job, and family careers.

Our final goal was to examine to what extent the impact of partnership dissolution on returning home differed by ethnicity (Hypothesis 5a) and whether this effect differed by gender (Hypothesis 5b). To facilitate interpretation, Table 4 presents 10 separate models of returning home for the five ethnic groups and for each gender. To formally report whether ethnic differences in the effect of partnership dynamics on returning home were statistically significant from the native Dutch (*p* < .05 in bold), we additionally undertook a pooled model with partnership dynamics interacted with ethnic origin and gender (not shown). A striking feature of Table 4 is the large odds ratios for union dissolution among all origin groups, but particularly among native Dutch women. There are two explanations for this. First, descriptive research in the Netherlands suggested that partnership dissolution is the most important reason for returning home (Wobma & de Graaf, 2010). Second, union dissolution implies that at least one partner moves, meaning that many union dissolutions occurred in the exact same month as returning home, even though partners may have stopped living together informally months prior. Because of the use of monthly data, partnership dissolution was a rare event over all person‐period observations (0.6%), but quite common at the moment of returning home (19.4%), resulting in large effect sizes.

**Table 4:** Odds Ratios From Discrete‐Time Event History Models of Returning to the Parental Home, by Gender and Origin Group

	Turkish	Moroccan	Surinamese	Antillean	Dutch
Men					
Stable unpartnered (ref.)	1.00	1.00	1.00	1.00	1.00
Stable cohabiting	0.02***	0.01***	0.03***	0.02***	0.03***
Stable married	**0.17** ***	**0.14** ***	**0.10** ***	0.06***	0.04***
New cohabiting	**1.08**	**0.19** ***	**0.49** ***	0.15***	0.09***
New marriage	**1.26**	**0.39** *	0.23***	0.01***	0.05***
Dissolution	**18.43** ***	**17.84** ***	26.78***	25.16***	27.92***
Women					
Stable unpartnered (ref.)	1.00	1.00	1.00	1.00	1.00
Stable cohabiting	**0.01** ***	**0.01** ***	0.04***	0.04***	0.03***
Stable married	0.06***	**0.05** ***	0.10***	0.10***	0.07***
New cohabiting	**0.49** ***	0.19***	**0.52** ***	**0.26** ***	0.09***
New marriage	0.19***	0.08***	0.18*	0.02***	0.08***
Dissolution	**19.79** ***	**21.14** ***	**28.14** ***	**27.47** ***	41.62***

*Note*. Odds ratios in bold format indicate a statistically significant difference from the native Dutch (*p* < .05) based on interaction terms between ethnicity and partnership dynamics in a pooled model for each gender (not shown). Baseline hazard function, mixed parentage, age at leaving home, economic activity, parenthood, educational level, urbanicity, calendar period, parental union status, and parental occupational status are held at their baseline values. ref. = reference.

* *p* < .05.

*** *p* < .001.

As can be seen in Table 4, the relationship between partnership dissolution and returning home was stronger for the native Dutch than for the Turkish and Moroccan second generation, supporting Hypothesis 5a. In line with Hypothesis 5b, this difference was larger among women; a three‐way interaction effect confirmed a significant difference (*p* < .01). We also found a significantly weaker effect of partnership dissolution for Surinamese and Antillean women than for native Dutch women, although the differences were smaller. There was no statistically significant difference between these latter origin groups among men, however. Some other partnership stages and transitions also differed between ethnic groups. Perhaps most noteworthy was the finding that the negative effect of entering either a cohabiting or married relationship on returning home was stronger for native Dutch men than for the Turkish and Moroccan men. In fact, among Turkish men we even observed positive effects of entering a cohabiting or married relationship on returning home, although the estimates were not statistically significant. This finding is consistent with the cultural tradition among Turks and Moroccans that the woman moves in with her parents‐in‐law after leaving the parental home (Koc, 2007).

## Discussion

Despite recent attention being paid to the increasing numbers of young adults returning to the parental home, this is the first European study to examine ethnic differences in the mechanisms underlying this behavior. We compared the second generation of the four main non‐Western migrant groups in the Netherlands (Turks, Moroccans, Surinamese, Antilleans) with native Dutch. Administrative micro data available from the Dutch population registers permitted a detailed investigation, avoiding some of the methodological problems frequently encountered using survey data (e.g., small sample sizes, panel attrition, left censoring). Drawing on theories of value transmission from parents to children, we expected an inclination toward collectivism over individualism among second‐generation youth, making them more likely to return home than native Dutch among whom (residential) independence is valued. Indeed, all second‐generation groups were found to be more likely to return home than native Dutch. Previous research indicated that differences between the values held by migrant children's parents and the society in which these children grow up might increase intergenerational tensions and conflict (Giguère et al., 2010; Lou et al., 2011). This finding has been used to explain why migrant youth leave home at significantly younger ages and more often to live alone independently than native Dutch youth (Zorlu & Mulder, 2011).

We might question how can we reconcile the findings in this article—that migrant youth are more likely to return home—with increased intergenerational conflict. We put forward two suggestions. First, if migrant children experience more conflict with their parents, they may be more likely to leave the parental home through impulse (Davanzo & Goldscheider, 1990). In such circumstances, the departure is unlikely to be a carefully planned decision, which may cause problems for the young adult to retain independent residence in the long term. Second, we might question the validity of the assumption made in previous work about intergenerational conflict driving early home leaving because it seems unlikely that young adults and their parents are happy to coreside once again when this has caused problems in the past. An alternative explanation for the early home leaving is that departure from the parental home provides an opportunity for migrant children to gain upward mobility both in terms of housing quality (Zorlu & van Gaalen, 2016) and moving to a location that provides better opportunities for enrollment in higher education and access to jobs (Zorlu & Mulder, 2010). If this latter explanation holds true, our results suggest that disadvantaged minority youth may find it difficult to “escape” from socioeconomically deprived areas, as many departures are not sustainable and are frequently reversed.

We also aimed to gain insights into the role of early life course stages and transitions in explaining ethnic group differences in returning home. Specifically, we investigated whether ethnic differences in returning home were related to differences in age at leaving home, economic activity, and partnership dynamics. All three were found to contribute to the association between ethnicity and returning home, but the second‐generation groups were still significantly more likely to return home when all other observed factors were controlled, suggesting that cultural factors (and other unobserved factors) also play an important role in returning home. Age at leaving the parental home and partnership status were found to contribute much more substantially to the ethnic differences in returning home than economic activity. Our analysis suggests that the higher likelihood of the native Dutch to live with a partner was strongly related to their higher ages at leaving the parental household. Thus, although the finding that young home‐leavers are more likely to return is partially an effect of age itself, for example, because (returned) coresidence with parents may be more acceptable at younger ages (Aassve et al., 2013; Stone et al., 2014), the age at leaving home effect operates to a large extent through subsequent partnership trajectories.

This article has provided a greater understanding about how partnership dynamics relate to returning home and how these differ by ethnic group. The relationship between union dissolution and returning home was strongest among native Dutch women. The tendency of women to move out after union dissolution has been linked to homeownership, where it is assumed that men are in an advantaged position in keeping the owner‐occupied home (Mulder, Ten Hengel, Latten, & Das, 2012). This argument does not, however, equally apply to second‐generation youth who are largely concentrated in social rented housing (Zorlu, Mulder, & van Gaalen, 2014). Indeed, recent research on residential mobility following union dissolution showed that ethnic‐minority men move out comparatively more often than is the case among native Dutch ex‐couples (Rooyackers et al., 2015). Although this potentially explains why the observed association between union dissolution and home‐returning is less pronounced among second‐generation women than among native Dutch women, it cannot explain why Turkish and Moroccan men are less likely than native Dutch men to rejoin the parental home after divorce or separation as well. On the contrary, given that Turkish and Moroccan men change residence more frequently after union dissolution than native Dutch men do, one would expect them to be more likely to return to the parental home as well. Based on qualitative research (Hooghiemstra, 2003; Sterckx & Bouw, 2005), we argue that part of the explanation may lie in parental disapproval of cohabitation and divorce among the parents of the Turkish and Moroccan second generation, making some of them reluctant to welcome their children back home after partnership dissolution. Overall, however, it appears that young adults from all ethnic groups tend to seek shelter with their parents when their partnership ends.

Despite the previously discussed advantages of population register data, they also entail some disadvantages that should be noted. First, administrative data contain no direct measures of cultural norms and values. We therefore interpreted the residual ethnic differences in home‐returning after controlling for other relevant factors as subcultural effects. Other unobserved characteristics (e.g., social networks, religiosity, intergenerational conflict) are likely to influence young adults' home‐returning choices. Because these characteristics differ across ethnicity, our results potentially overestimate the importance of ethnic factors. Second, partnership dynamics are more complex than can be captured in population registers. Many young adults who do not live in a coresidential union do have a nonresidential partner, as currently almost 40% of the Dutch population aged 18 to 30 years is in an Living Apart Together (LAT) relationship (Statistics Netherlands, 2015). These nonresidential partners may serve as alternative sources of housing support if necessary, making a return to the parental home less likely. Third, although population register data provide information on the young adults' living arrangements after leaving the parental home, the reasons for leaving cannot be elucidated. Previous research shows that reasons for leaving home are strongly related to the likelihood of returning home (Goldscheider & Goldscheider, 1998). In view of these data limitations, future research may strongly benefit from combining register data with large‐scale survey data. Unfortunately, as of yet there are no survey data available that are suitable for these purposes.

Finally, the use of monthly data can be considered a strength as well as a limitation of this study. In contrast to previous research on returning home using annual or even biennial data (e.g., South & Lei, 2015; Stone et al., 2014), our data help identify the time ordering of events more accurately. Moreover, data with larger time intervals may miss short‐term moves entirely. At the same time, however, certain life course transitions, such as losing a job, can have a delayed or lagged effect on returning home. Many people can fall back on temporary unemployment benefits after losing their job, but once these run out people may be forced to return to live with their parents. Similarly, a person who becomes unemployed may remain in his or her current residence until the end of the tenancy contract and then return home, which might be several months later. Although sensitivity analysis using larger time intervals provided substantially similar results, it remains a challenge for future research and data collection initiatives to allow an examination of all the complexities and intricacies related to the dynamics of returning home.

## Note

This work is part of and financed by the European Research Council, starting grant project (no. 26.38.29) “Families of Migrant Origin: A Life Course Perspective” (FaMiLife) awarded to Helga A.G. de Valk. Ann Berrington's contribution is supported by the Economic and Social Research Council (ESRC) Centre for Population Change (Grant ES/K007394/1). The authors gratefully acknowledge the valuable comments and suggestions of Helga de Valk. Finally, the authors thank Statistics Netherlands for data preparation and providing the opportunity to let the first author work on site at their premises.
